# The Potential Role of Extracorporeal Cytokine Removal in Hemodynamic Stabilization in Hyperinflammatory Shock

**DOI:** 10.3390/biomedicines9070768

**Published:** 2021-07-01

**Authors:** Fatime Hawchar, Cristina Rao, Ali Akil, Yatin Mehta, Christopher Rugg, Joerg Scheier, Harriet Adamson, Efthymios Deliargyris, Zsolt Molnar

**Affiliations:** 1 Department of Anaesthesiology and Intensive Therapy, University of Szeged, 6 Semmelweis Str., H-6725 Szeged, Hungary; hawchar.fatime@med.u-szeged.hu; 2 Cytosorbents Europe GmbH, Müggelseedamm 131, 12587 Berlin, Germany; cristina.rao@cytosorbents.com (C.R.); joerg.scheier@cytosorbents.com (J.S.); harriet.adamson@cytosorbents.com (H.A.); 3 Department of Thoracic Surgery and Lung Support, Klinikum Ibbenbueren, Grosse Strasse 41, 49477 Ibbenbueren, Germany; a.akil@klinikum-ibbenbueren.de; 4 Institute of Critical Care and Anesthesiology, Medanta the Medicity, CH Baktawar Singh Rd, Gurugram 122001, Haryana, India; yatin.mehta@medanta.org; 5 Department of Anesthesiology and Critical Care Medicine, Medical University of Innsbruck, Anichstrasse 35, 6020 Innsbruck, Austria; christopher.rugg@tirol-kliniken.at; 6 Cytosorbents Corporation, 7 Deer Park Drive Suite K, Monmouth Junction, NJ 08852, USA; edeliargyris@cytosorbents.com; 7 Institute for Translational Medicine, School of Medicine, University of Pécs, Szigeti Str. 12, H-7624 Pécs, Hungary; 8 Department of Anaesthesiology and Intensive Therapy and Pain Management, Poznan University for Medical Sciences, 61-701 Poznan, Poland; 9 Department of Anaesthesiology and Intensive Therapy, Semmelweis University, 1085 Budapest, Hungary

**Keywords:** shock, hemodynamic stabilization, hemoadsorption, cytosorb therapy, hypeinflammation, decatecholaminization

## Abstract

Hemodynamic instability due to dysregulated host response is a life-threatening condition requiring vasopressors and vital organ support. Hemoadsorption with Cytosorb has proven to be effective in reducing cytokines and possibly in attenuating the devastating effects of the cytokine storm originating from the immune over-response to the initial insult. We reviewed the PubMed database to assess evidence of the impact of Cytosorb on norepinephrine needs in the critically ill. We further analyzed those studies including data on control cohorts in a comparative pooled analysis, defining a treatment effect as the standardized mean differences in relative reductions in vasopressor dosage at 24 h. The literature search returned 33 eligible studies. We found evidence of a significant reduction in norepinephrine requirement after treatment: median before, 0.55 (IQR: 0.39–0.90); after, 0.09 (0.00–0.25) μg/kg/min, *p* < 0.001. The pooled effect size at 24 h was large, though characterized by high heterogeneity. In light of the importance of a quick resolution of hemodynamic instability in the critically ill, further research is encouraged to enrich knowledge on the potentials of the therapy.

## 1. Introduction

Critical illness can occur due to several different etiologies, though commonly ending in similar trajectories. Regardless of the initiating insult, vital organ functions, not necessarily affected primarily, fall victim to a dysregulated host response [1]. Systemic hyperinflammation and the cytokine storm play a key role in this pathophysiology [2]. The cytokine storm originating from the immune over-response determines impairment of the vascular tone and systemic vasodilation, which manifests as hemodynamic instability. In its most serious form as vasoplegic circulatory shock, hemodynamic instability can be life-threatening; consequently, reversing shock as soon as possible is a lifesaving measure of utmost importance to avoid the devastating effects of hypoxemic organ damage [3]. CytoSorb(CytoSorbents, Monmouth Junction, NJ, USA) is a CE mark-approved extracorporeal therapy fort he adsorption and removal of small and middle-sized molecules in the blood (the adsorption spectrum is 5–60 kDa). The therapy has the potential to effectively remove cytokines originating from the cytokine storm [4,5], and thus can mitigate systemic hyperinflammation, contribute to early shock reversal, and last but not least, improve clinical outcomes. This systematic review will attempt to objectively assess the ability of CytoSorbtherapy (hereafter Cytosorb) to reduce the need of vasopressor support and to reverse vasoplegic shock, based on the available published literature.

### 1.1. Physiology of Shock

Shock is currently defined by the task force from the European Society of Intensive Care Medicine (ESICM) as a “life-threatening, generalized form of acute circulatory failure associated with inadequate oxygen utilization by the cells” [6]. Generally, this means an imbalance between oxygen delivery (DO_2_) and oxygen consumption (VO_2_). DO_2_ depends on cardiac output (CO) and the arterial oxygen content (CaO_2_), and VO_2_ depends on mixed venous oxygen content (SvO_2_):

DO_2_ =CO × CaO_2_ CaO_2_ = Hb × 1.34 × SaO_2_ +0.003 × PaO_2_ DO_2_ =CO × (Hb × 1.34 × SaO_2_ +0.003 × PaO_2_) VO_2_ = CO × (CaO_2_ − CvO_2_) VO_2_ = CO × [(Hb × 1.34 × SaO_2_ + 0.003 × PaO_2_) − (Hb × 1.34 × SvO_2_ +0.003 × PvO_2_), (1)

where DO_2_ is oxygen delivery; CO is cardiac output; Hb is hemoglobin; SaO_2_ is arterial oxygen saturation; PaO_2_ is partial pressure of oxygen in the arterial blood; CaO_2_ is arterial oxygen content; VO_2_ is oxygen consumption; SvO_2_ is mixed venous oxygen saturation; CvO_2_ is mixed venous oxygen content; PvO_2_ is mixed venous oxygen tension.

Adequate oxygen supply is paramount for preserving organ viability and is dependent on adequate tissue perfusion. The latter is commonly assessed by mean arterial pressure (MAP), which is mainly determined by vascular tone (systemic vascular resistance—SVR). The relationship between SVR, MAP, central venous pressure (CVP) and CO is described below, based on Ohm’s law:

(2) $$\mathrm{SVR}=\frac{\mathrm{MAP}-\mathrm{CVP}}{\mathrm{CO}}$$

**Figure 1:**
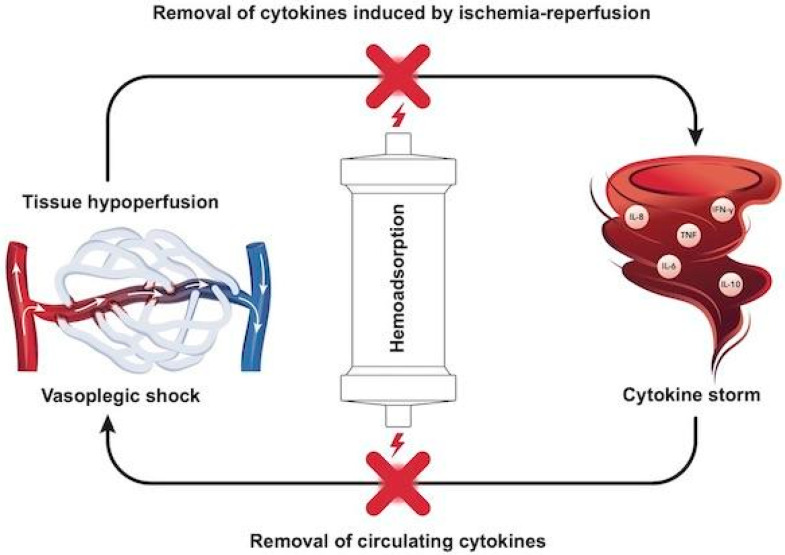
The vicious circle of hyperinflammation. Circulating cytokines can induce vasodilatation leading to arteriovenous shunting in the microcirculation and eventually vasoplegic shock. Hypoperfused tissues may further amplify the effects of cytokine storm byconcomittantly triggering an immune response, potentially leading to ischemia–reperfusion injury. Hemoadsorption can potentially attenuate this vicious circle and protect the tissues from this onslaught by removing circulating cytokines and those released after tissue injury.

With the help of these formulae, the mechanisms of the various shock types can be easily understood [7]. Loss of vascular tone (i.e., sepsis, hyperinflammation) results in low SVR, low MAP and preload deficit (i.e., vasoplegic shock). The different underlying mechanisms of hemodynamic instability also correspond to potential therapeutic options to be targeted, including fluids, inotropes, oxygen supplementation, and vasopressors to increase vascular tone, hence tissue perfusion. The differential diagnosis of hemodynamic instability or shock requires a skilled assessment of the complete clinical picture, which ranges from a simple measurement of vital signs such as heart rate and blood pressure, to complex, advanced hemodynamic monitoring [8]. Only after comprehensive assessment can the clinician determine the underlying pathophysiologic mechanisms and decide upon the best course of action and the right combination of interventions for each individual patient. The connection of inflammatory response–vasoplegia–tissue hypoperfusion–cytokine removal is depicted in Figure 1.

Different types of shock require different management strategies. However, hemodynamic stabilization always represents a main goal due to its role in reestablishing adequate aerobic metabolism in the cells and tissues, and in regaining control over the oxygen debt. Oxygen debt also accumulates during the resuscitation period, suggesting that shorter resuscitation times translate into lesser oxygen debts. Experimental studies suggest that both the severity and duration of hemodynamic instability are associated with poor outcomes [9]. 

### 1.2. Hyperinflammation and Vasoplegic Shock

Vasoplegic shock in the setting of hyperinflammation is a particularly challenging scenario. In the past, the clinical and biochemical characteristics of vasoplegic shock were often defined within the domain of “septic shock”. However, similar features are also observed in non-infective inflammatory states, such as in acute necrotizing pancreatitis, after major trauma, major surgery, and in other conditions without an obvious infectious component. In order to better capture the underlying pathophysiology, an updated definition was derived via expert consensus [1,10]. The main message of the updated definition is that the pathophysiological process is fundamentally the same regardless of the provoking injury/insult, and is mainly determined by the host response. This was in large part supported by the very important discovery that both pathogen- and damage-associated molecular patterns (PAMPs and DAMPs) can trigger a septic shock-like clinical picture [9]. Interestingly, very similar observations were made many years before by Sir William Osler in the context of bacterial infections [11], and Hans Janos Selye in the context of stress [12]. Based on our current understanding, the term “hyperinflammatory shock” is preferred over “septic shock”, since it describes the pathophysiology more accurately and is applicable to both infectious and non-infectious etiologies.

The term “refractory shock” is commonly used to describe the most severe cases of hyperinflammatory shock. Although there is no clear consensus as to the exact definition for refractory shock, it is generally intended as shock persisting for more than 6 h despite initiation of full standard therapy, and is indicated by the following:

- Elevated lactate levels (>2.9 mmol/L) [13];
- High norepinephrine (NE) requirements (>0.3 µg/kg/min) [13].

### 1.3. Shock and Shock Reversal 

Up to one-third of patients admitted to the intensive care unit (ICU) are in circulatory shock [14]. Septic shock is by far the most frequent type of shock (62%) and carries a very high mortality ranging from 40 to 80% [15]. As noted already, the expert community now recommends the terms “hyperinflammatory” or “vasoplegic” shock over “septic shock” to better reflect the underlying pathophysiology of a “dysregulated host response” [1,5]. Several studies have demonstrated that both the severity and duration of hemodynamic instability (i.e., hypotension, low CO) are associated with poor outcomes [16,17]. Accordingly, current Surviving Sepsis Guidelines recommend early and aggressive resuscitation within the first hours of the onset of sepsis and septic shock [18]. Fluid resuscitation is usually the first step in the resuscitation algorithm. However, especially in vasoplegic shock, which is characterized by low SVR and consequently low diastolic blood pressure, a fluid challenge alone is often insufficient to improve tissue perfusion [19,20]. Starting vasopressors to increase the vascular tone and SVR together with fluid resuscitation in severe cases has a strong pathophysiological rationale. Clinical studies have shown that delaying the introduction of these therapeutic measures is associated with increased risk of death [21,22].

Vasopressors exert their effect by either mimicking the effect of the sympathetic nervous system (sympathomimetic amines) or by raising extracellular ionized calcium concentrations (calcium chloride). Sympathomimetic amines can be divided into either catecholamines or non-catecholamines. Commonly used catecholamines with a prominent agonist activity include epinephrine (also known as adrenaline), norepinephrine (noradrenaline) and dopamine. Norepinephrine is recommended as first line treatment of septic shock by the Surviving Sepsis campaign, but the combined use of vasopressors including both vasopressin and norepinephrine is also suggested to limit adrenoceptor desensitization due to sympathetic hyperactivation [23,24]. 

In patients with severe hyperinflammatory shock, neither vasopressors nor fluid resuscitation are effective in quickly reversing shock. Given the pathophysiological background, cytokine removal through hemoadsorption might be beneficial for patients showing resistance to resuscitation, i.e., not stabilized after 6 h of resuscitation and organ support. Cytokine removal can attenuate hyperinflammation and hence vasoplegia, leading to quicker hemodynamic stabilization and shock reversal. The most frequently used criteria to define shock reversal include normalization of serum lactate (<2.2 mmol/L) coupled with a significant (≥90%) reduction in norepinephrine dose requirements [13,25].

## 2. Objectives

This review is aimed at assessing the effect of hemoadsorption with Cytosorb on the need for vasopressor support. We achieved this by analyzing norepinephrine dose requirements before and after treatment with Cytosorb in patients with vasoplegic shock of various etiologies. 

## 3. Materials and Methods

A literature search was conducted in PubMed (https://pubmed.ncbi.nlm.nih.gov/, accessed on 10 March 2021), with the last update on 10 March 2021. The key search word “Cytosorb” was used for the search. We did not apply any restrictions in terms of study design. We selected articles reporting on requirements of norepinephrine to analyze whether a reduction in vasopressor support could be observed. We included only studies recording and reporting norepinephrine doses in the microgram per kilogram per minute (µg/kg/min) measurement scale, and where the vasopressor dose was measured before and after treatment with Cytosorb. We summarized data from studies to assess the variation in vasopressor needs before and after treatment with Cytosorb, without considering the heterogeneity existing among different sources. In addition to the descriptive study, a pooled comparative analysis was conducted for studies including data on control cohorts. The effect size was expressed as the standardized mean difference of the relative reduction in the need for vasopressor support from baseline to 24 h.The analysis of data was conducted using Microsoft Excel version 16 (Microsoft Corporation. 2019. Redmond, WA, USA) and STATA statistical software, release 16 (StataCorp LLC. 2019. College Station, TX, USA).

## 4. Results

Out of the 163 clinical articles available in PubMed, 58 were identified that mentioned “catecholamines and/or vasopressors”. In total, 25 papers were excluded; 12 because of non-comparability of the measurement scales [25,26,27,28,29,30,31,32,33,34,35,36]; 4 because the type and dose of vasopressors were not specified [37,38,39,40]; 1 that reported combined norepinephrine and epinephrine doses [41]; 1 that only reported on patients that had survived [42]; and 7 where there were no measurements pre- and post-adsorber use in the same patient [43,44,45,46,47,48,49]. The remaining 33 articles were summarized without considering different study designs or duration of treatment. Overall, data on 353 patients treated with Cytosorb were collected. 

Table A1 in Appendix A depicts details from articles included in our review [13,50,51,52,53,54,55,56,57,58,59,60,61,62,63,64,65,66,67,68,69,70,71,72,73,74,75,76,77,78,79,80,81]. From these papers, four were selected for a pooled comparative analysis due to their inclusion of both Cytosorb and a control cohort [64,75,77,79]. 

The highest and the lowest administered doses of norepinephrine for each day were reported at 24, 48, 72 or 96 h after the start of Cytosorb treatment, depending on the specific study. We assumed as the pre-Cytosorb value the dose administered before the start of the therapy or the highest dose recorded during the first 24 h before the start of hemoadsorption, depending on data availability, and the post-Cytosorb value as the lowest dose of norepinephrine administered and recorded during the last reported day. We assumed the last available norepinephrine dose measurement to be at the end of Cytosorb therapy for all patients in all studies analyzed. However, we are aware of at least one study [13] wherein the norepinephrine dose was measured and recorded only during the first day, while the therapy was used for an additional two days. We still used the lowest available dose to determine norepinephrine requirements after hemoadsorption treatment. 

**Figure 2:**
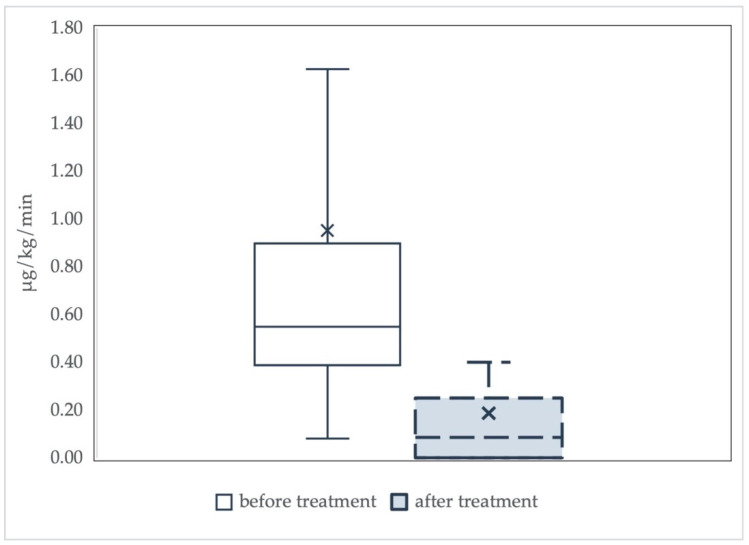
Norepinephrine requirements before and after treatment with Cytosorb. Data are summarized as boxplots. The “x” in the box represents the mean value. There is a significant decline in median norepinephrine requirements before and after hemoadsorption with Cytosorb (from 0.55 (0.39–0.9) µg/kg/min to 0.09 (0.0–0.25) µg/kg/min, *p* < 0.001).

The descriptive analysis comprised 21 case reports, 11 case series and one randomized trial, and did not consider differences in the number of adsorbers used or the duration of treatment sessions. The results of the analysis are summarized in Figure 2.

In 14 articles, including three case series, norepinephrine was weaned off after treatment with Cytosorb. Norepinephrine dosage was higher than 0.5 μg/kg/min at the end of the treatment with Cytosorb in one case report [74], and in two case series [12,78]. The median dosage of norepinephrine required decreased by a full order of magnitude at the end of Cytosorb therapy. Overall, the available evidence shows that the norepinephrine dose requirements were markedly lower after Cytosorb treatment. 

### 4.1. Analysis of Studies with Control Cohorts

Four of the articles reported norepinephrine requirements in patients treated with Cytosorb as well as in a control cohort not treated with Cytosorb (Table 1).

**Figure 3:**
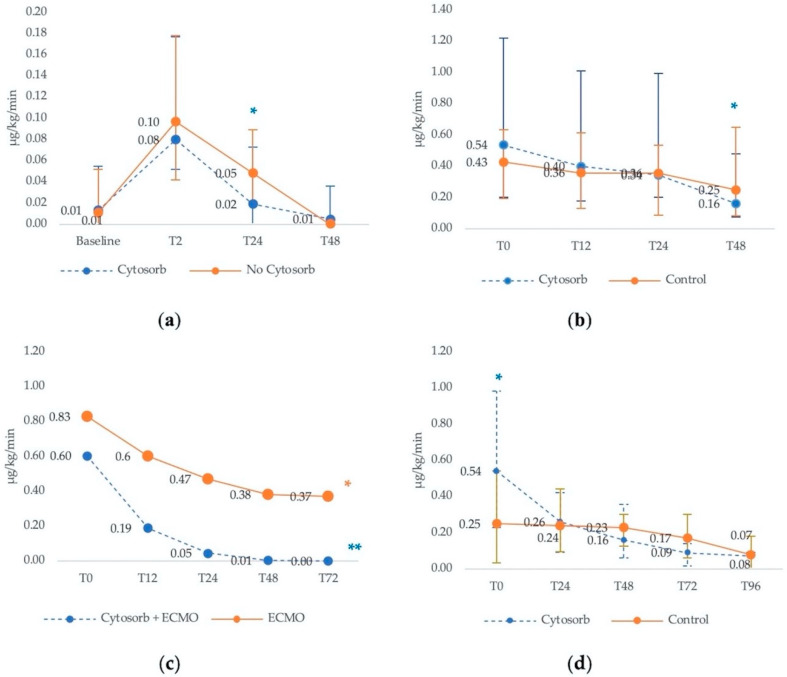
Median vasopressor therapy requirements in Cytosorb and control cohorts. (**a**) Median vasopressor therapy requirements in aortic surgery patients. Based on Mehta et al. [77]. * *p* < 0.05 for NE dose of Cytosorb vs. no Cytosorb at T24. (**b**) Median vasopressor therapy requirements in septic patients. Based on Hawchar et al. [64]. T_0_ is measured right after inclusion (control) or start of hemoadsorption. T_12_, T_24_ and T_48_ were measured 12, 24 and 48 h later. * *p* < 0.05 vs. T_0_ in the Cytosorb group. (**c**) Mean vasopressor therapy requirements in patient with pneumonia-derived sepsis. Based on Akil et al. [29]. Timepoints represent hours after the initial dose administered at the entrance into the ICU. * *p* = 0.05 at T_48_ and T_72_ in the ECMO group. ** *p* < 0.005 at T_12_, T_24_, T_48_ and T_72_ in the Cytosorb group. (**d**) Median vasopressor therapy requirements in septic shock patients requiring CRRT. Based on Rugg et al. [79]. Baseline is defined as the day of Cytosorb mounting in the treatment group. Data are presented as median and interquartile ranges. * *p* = 0.014 as compared to baseline. For explanation see text.

Mehta et al. [77] compared outcomes between patients undergoing aortic surgery with Cytosorb installed in the cardiopulmonary bypass circuit with patients undergoing conventional surgery without Cytosorb adsorber. At baseline, after the induction of anesthesia, there was no difference in the median dosage of norepinephrine in the Cytosorb or control groups, and vasopressor requirements remained similar at 2 h after discontinuation of CPB (Figure 3a). However, by 24 h after surgery, the median need for vasopressor dose was significantly lower in the Cytosorb group compared to controls. After 48 h, all patients were either weaned off or only had minimal vasopressor requirements. 

The remaining three articles included septic shock patients, and in all of these the use of Cytosorb was associated with a quicker reduction in norepinephrine needs [64,75]. 

In Hawchar et al. [64], 20 patients with early onset sepsis were randomly assigned to receive either Cytosorb (*n* = 10) or standard care (*n* = 10). All patients were mechanically ventilated and on hemodynamic monitoring-guided norepinephrine. Although norepinephrine requirements declined in both groups over time, the decline after 48 h was only significant in the Cytosorb group. Specifically, in the Cytosorb group, norepinephrine doses declined at a steady rate and significantly over 48 h (Figure 3b). In the control group, lesser and slower declines in norepinephrine requirements over time were observed, with the overall trend not being significant. The mean change (Δ) in norepineprine requirements between baseline and 48 h was also significantly greater in the Cytosorb group (0.67 μg/kg/min vs. 0.10 μg/kg/min; *p* = 0.047). 

In another study investigating the role of Cytosorb in septic patients, Akil and colleagues [75] prospectively compared 13 patients who developed acute respiratory distress syndrome (ARDS) from pneumonia-derived sepsis and were treated with veno-venous extracorporeal membrane oxygenation (ECMO) plus Cytosorb to a historical cohort of 7 pulmonary sepsis patients treated with ECMO alone. At the time of admission to the ICU, norepinephrine dose was slightly lower in the Cytosorb group compared to controls, but both patient groups required high vasopressor support (Figure 3c). 

Although reductions in vasopressor requirements were observed in both groups, the decline in the hemoadsorption group was more rapid and more pronounced. Specifically, median norepinephrine dose was significantly reduced after 12, 24 and 48 h of treatment compared to the initial dose required at the time of admission in the ICU. After 72 h, none of the Cytosorb patients required norepinephrine, while in the control group, high norepinephrine doses were still needed after 12, 24, 48 and 72 h after admission to the ICU (Figure 3c).

Finally, in a retrospective study of Cytosorb in septic shock patients, Rugg and colleagues [79] compared the catecholamine requirements of 42 septic shock patients treated with Cytosorb and continuous renal replacement therapy (CRRT) to a genetic-matched control of 42 patients receiving CRRT alone. Baseline catecholamine requirements were significantly lower in the control group compared with the Cytosorb group, suggesting that the latter patients were sicker. However, within 24 h of Cytosorb initiation, norepinephrine doses were halved, while no change was seen in the control group. By 96 h, the vasopressor requirements were similar in both groups, but the overall reduction in patients not receiving Cytosorb therapy was modest and very slow (Figure 3d).

### 4.2. Pooled Analysis 

We pooled together the results from the four studies with control cohorts to estimate the effect size of the benefit associated with the use of Cytosorb treatment, expressed in terms of reduced need of vasopressor support at 24 h. 

The meta-analysis was run on STATA 16 [82] using the meta command. The effect size was estimated as the standardized mean difference of the relative reduction in the need for vasopressor support from baseline to 24 h. We used Hedge’s g statistical method, which is preferred for estimates on small samples. The effect size according to Hedge’s g is interpreted following a rule of thumb:

- Small effect = 0.2;
- Medium effect = 0.5;
- Large effect = 0.8.

**Figure 4:**
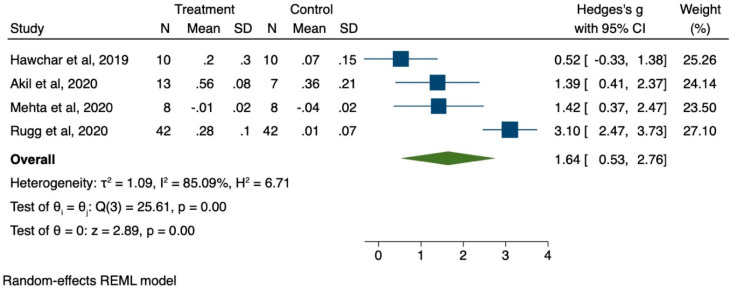
Forest plot for efficacy of CS therapy to reduce NE requirements at 24 h.

Figure 4 below summarizes the results of the pooled analysis. 

The pooled effect size at 24 h was large and statistically significant. Despite the consistency in the direction of the treatment effect, the I^2^ statistic suggests a high degree of heterogeneity in the size of the treatment effect between the studies. 

## 5. Discussion 

Despite advancements in critical care medicine, critical illness and hyperinflammatory shock are still characterized by high mortality all over the world, and create a huge demand for advancements in critical care. The available therapeutic strategies aim at supporting the impaired organ function and at re-establishing hemodynamic stability.

Fluid resuscitation and vasopressor therapies represent important first-line options in these patients. However, both excessive fluid administration and high doses or prolonged usage of vasopressors can lead to potential patient harm [19]. First, fluid overload can trigger respiratory and cardiac strain, both manifesting in worsening hypoxemia and myocardial ischemia. For this reason, fluid “*de-resuscitation*” should be aggressively pursued after hemodynamic stabilization is established. Second, vasopressors may cause vasoconstriction in the arterioles, and thus decrease microvascular perfusion, an effect demonstrated in both healthy subjects [83] and critically ill patients [84,85]. The potentially serious adverse effects of high-dose vasopressor administration include digital ischemia, tachyarrhythmias, facilitation of bacterial growth, and compromised host resistance to bacteria [86] (Table 2). 

Importantly, several retrospective studies have concluded that the prolonged use of high-dose norepinephrine is associated with poor outcomes, and is also a strong predictor of death [87,88]. Although one could argue that high-dose vasopressors are simply a surrogate marker of disease severity in these patients, these results suggest that reducing the need for vasopressor support in terms of both time and dosage could be beneficial for patients.

These findings emphasize the importance of shock reversal with concomitant “*de-catecholamisation*”, to be performed as quickly as possible [89,90,91,92]. 

Cytosorb is a European CE-marked therapy able to adsorb and thus remove cytokines from the blood, attenuating the devastating effects of the cytokine storm. In this review, we have found a significant decline in vasopressor support requirements after treatment with hemoadsorption in the critically ill. In addition, based on a pooled analysis of studies including data on control cohorts, we have found evidence of a large treatment effect of the therapy at 24 h from baseline. This finding was characterized by large heterogeneity, indicating variability among studies. 

The paper has several limitations. For example, we described the change in norepinephrine dose requirements in the Cytosorb population by including all types of published articles. In fact, data were generated from extremely heterogeneous sources with no standardization in regard to patients (besides observational studies, 25 single case reports), pathophysiology, clinical circumstances and time frame of observation. Furthermore, we could not take into consideration the number of adsorbers used during the observational period considered, nor the duration of use of each hemoadsorption cartridge, or whether the therapy continued after the last available vasopressor dose measurement. Another limitation is that we only evaluated one hemoadsorption device. Although there are other hemoadsorbers on the market (Jafron, Jafron Biomedical Co., Guangdong, China; Biosky, Biosun Medical Technology Co., Foshan, China), published data are extremely scarce in general, and none are available in the current context of hemodynamic stabilization, as was nicely summarized in a recently published review by Krenn and Stelzer [93]. Finally, we cannot make any comments on either shock reversal—as data on metabolic changes are missing—or on other beneficial effects on outcome, including survival. These issues have to be raised and answered in large prospective randomized studies.

Nevertheless, the results of this analysis encourage more research to be done in order to assess the potential use of the therapy in accelerating shock reversal and improving outcomes in the critically ill. Finally, the topics discussed here also provide food for thought on the need to better investigate the benefits derived from early control of the escalating cytokine storm in pre-hyperinflammatory states.

## 6. Conclusions

Intensive care medicine is one of the most dynamic fields in medicine, and is constantly evolving in terms of both disease state understanding and treatment advancements. The new definition of sepsis emphasizes the importance of “dysregulated immune response”, and other new terms increasingly used in this clinical setting include: hyperinflammation, cytokine storm, vasoplegic shock, refractory shock and shock reversal. These concepts more accurately reflect the improved understanding of the underlying pathophysiologic mechanisms, and as such could also help define priorities and clinical endpoints in the design of future clinical trials. Such an approach may also address the concerns of many experts that mortality may no longer be the only appropriate primary endpoint for clinical trials in this setting [94,95]. This is supported by recent trials in sepsis, applying composite clinical outcomes such as “vasopressor and mechanical ventilation-free days” as primary endpoints instead of mortality [96,97]. The current paper has summarized the available data, which indicate the important contribution of early hemoadsorption in achieving rapid hemodynamic stabilization in patients with refractory vasoplegic shock. 

## Figures and Tables

**Table 1:** Studies with Cytosorb and control cohorts.

Study	Design	Indication	Cytosorb, *n*	Control, *n*	Total
Mehta et al. [77]	Observational	Aortic surgery	8	8	16
Hawchar et al. [64]	Randomized	Septic shock	10	10	20
Akil et al. [75]	Observational	Septic shock	13	7	20
Rugg et al. [79]	Observational	Septic shock	42	42	84
Total	-	-	73	67	140

Summary results for the selected studies are depicted in Figure 3.

**Table 2:** Studies with CytoSorb and control cohorts.

Vasopressor	Dose	Potential Side Effects
Norepinephrine (noradrenaline)	0.05–0.1 mcg/kg/min	Acute glaucoma; anxiety; arrhythmias; asthenia; cardiomyopathy; confusion; dyspnea; extravasation necrosis; gangrene; headache; heart failure; hypovolemia; hypoxia; injection site necrosis; insomnia; ischemia; increased myocardial contractility; nausea; palpitations; peripheral ischemia; psychotic disorder; respiratory failure; tremor; urinary retention; vomiting
Dopamine	Up to 20 mcg/kg/min	Angina pectoris; anxiety; arrhythmias; azotemia; cardiac conduction disorder; dyspnea; gangrene; headache; hypertension; mydriasis; nausea; palpitations; piloerection; polyuria; tremor; vasoconstriction; vomiting
Epinephrine (adrenaline)	0.01–0.1 mcg/kg/min	Angina pectoris; angle closure glaucoma; anxiety; appetite decreased; arrhythmias; asthenia; CNS; hemorrhage; confusion; dizziness; dry mouth; dyspnea; headache; hepatic necrosis; hyperglycemia; hyperhidrosis; hypersalivation; hypertension (increased risk of cerebral hemorrhage); hypokalemia; injection site necrosis; insomnia; intestinal necrosis; metabolic acidosis; mydriasis; myocardial infarction; nausea; pallor; palpitations; peripheral coldness; psychosis; pulmonary edema (on excessive dosage or extreme sensitivity); renal necrosis; soft tissue necrosis; tremor; urinary disorders; vomiting
Vasopressin	0.01–0.07 units/min	Abdominal pain; angina pectoris; bronchospasm; cardiac arrest; chest pain; diarrhea; pain; flatulence; fluid imbalance; gangrene; headache; hyperhidrosis; hypertension; musculoskeletal chest pain; nausea; pallor; peripheral ischemia; tremor; urticaria; vomiting; vertigo
Dobutamine	2.5–10 mcg/kg/min	Arrhythmias; bronchospasm; chest pain; dyspnea; eosinophilia; fever; headache; localized inflammation; ischemic heart disease; nausea; palpitations; platelet aggregation inhibition (on prolonged administration); skin reactions; urinary urgency; vasoconstriction

Please note, the depicted doses refer to the most frequently reported values and do not represent recommendations.

## Data Availability

The data presented in this study are available on request from the corresponding author.

## Appendix A

Table A1 reports details of studies included in this review.

**Table A1:** Details of included studies.

Reference	Type of Patients	Num of pts	Avg. Num of Adsorbers	Time Frame for Use	End Points	Results
Mitzner et al. [50]	Septic shock	1	1	24 h	None stated	86.67% reduction in norepinephrine use in 24 h
Hetz et al. [51]	Septic Shock	1	3	24 h each	None stated	83.05% reduction in norepinephrine use in first 24 h
Frimmel et al. [52]	Viral shock, ALF	1	1	24 h	None stated	58.33% reduction in norepinephrine use in 24 h
Hinz et al. [53]	Septic shock, ALF	1	3	1. 24 h, 2. 2.6 h, 3. 5 days later 24 h	None stated	76.25% reduction over 3 days
Traegar et al. [54]	Septic shock, ARDS	1	3	1. 20 h, 2. 35 h, 3. 29 h	None stated	50% increase in norepinephrine use after 1st treatment, 66.6% decrease on 2nd treatment, 100% decrease day 3.
Van der Linde et al. [55]	Septic shock, ARDS	1	1	24 h	None stated	100% reduction in norepinephrine use
Marek et al. [56]	Cardiogenic shock, post cardiac arrest	1	4	4 × 24 h	None stated	36% reduction in norepinephrine on day 1, 31% reduction day 2, 34% reduction day 3
Friesecke et al. [13]	Refractory septic shock	20	3	3 × 24 h	Primary endpoint; change in norepinephrine requirement after 6 and 12 h of treatment with CytoSorb compared with start	51% reduction in norepinephrine in first 24 h
Napp et al. [57]	Acute poisoning/intoxication	1	2	2 × 24 h	None stated	50% reduction in norepinephrine day 1, 100% reduction day 2
Steltzer et al. [58]	Septic Shock	1	6	12 h each	None stated	36.6% reduction in norepinephrine requirement on day 1
Eid et al. [59]	Necrotizing fasciitis	1	2	2 × 24 h	None stated	95% reduction in norepinephrine requirements over 48 h
Nemeth et al. [60]	Cardiac transplant	24	24	Intra-op use	Primary outcome: hemodynamic stability and vasopressor demand during first 48 h post-op; magnitude of postoperative inflammatory response (PCT and CRP)	57% reduction in norepinephrine requirements on day 1
Nemeth et al. [61]	Septic shock, cardiogenic shock	1	1	1 × 24 h	None stated	100% reduction in norepinephrine in 24 h
Dogan et al. [62]	Cardiogenic shock	1	9	9 × 24 h with 23 day pause	None stated	61.54% reduction in norepinephrine on day 1, 12.5% increase day 2, 33.3% reduction day 3, 8.33% reduction day 4
Leonardis et al. [63]	Pneumococcal Sepsis Pt 1	1	4	Over 68 h	None stated	Initial increase in norepinephrine on day 1 (150%), 20% decrease day 2, 50% decrease day3, 75% decrease day 4
Leonardis et al. [63]	Meningococcal Sepsis Pt 3	1	2	Over 32 h	None stated	60% decrease in norepinephrine on day 1 and 100% decrease on day 2
Leonardis et al. [63]	Meningococcal Sepsis Pt 4	1	4	4 × 24 h	None stated	20% decrease on day on of norepinephrine, 37.5% decrease day 2, 20% decrease day 3, 100% decrease day 4
Leonardis et al. [63]	Meningococcal Sepsis Pt 4	1	4	4 × 24 h	None stated	20% decrease on day on of norepinephrine, 37.5% decrease day 2, 20% decrease day 3, 100% decrease day 4
Hawchar et al. [64]	Septic shock	10	1	1 × 24 h	Organ dysfunction and inflammatory response	37% reduction seen in norepinephrine on day 1
Kuhne et al. [65]	Intra-op cardiac surgery	10	1	Intra-op use only	None stated	37.5% reduction in norepinephrine use
Kuhne et al. [65]	Intra- and post-op cardiac Surgery	10	1	Intra- and post-op use for 72 h	None stated	75% reduction in norepinephrine use
Poli et al. [66]	Septic shock	1	4	1 × 9 h, 3 × 24 h	None stated	0% reduction norepinephrine use day 1, 80% day 2, 50% on day 3, 100% on day 4
Perez et al. [67]	Pediatric cardiogenic shock	1	1	1 × 72 h	None stated	27% reduction norepinephrine use day 1, 45% reduction day 2, 14% reduction day 3, 100% reduction day 4
Frimmel et al. [68]	Septic shock, ALF, HLH. Pt 1	1	1	24 h	None stated	58% reduction in norepinephrine
Frimmel et al. [68]	Septic shock, HLH Pt 2	1	2	2 × 24 h	None stated	0% reduction in norepinephrine on day 1, 28.6% reduction on day 2, 100% reduction day 3
De Schryven et al. [69]	Acute poisoning/intoxication	1	1	Not stated	None stated	92.3 reduction in norepinephrine over 3 days
Klinkmann et al. [70]	Fungal sepsis	1	1	20 h	None stated	78% reduction in norepinephrine use on first day
Dimski et al. [71]	Septic shock	11	1	1 × 24 h	Primary endpoint: feasibility of combined CytoSorb/CVVHD treatment with RCA	66% reduction in norepinephrine use on first day
Traegar at al. [72]	Cardiogenic shock post cardiac surgery	23	2	Various lengths of time	None stated	87% reduction in norepinephrine use on day 1, 80% reduction on day 2
Stahl et al. [73]	Cytokine release syndrome	1	5	Various lengths of time	None stated	47% decrease in norepinephrine on day 1, 57% reduction on day 2
Dilken et al. [74]	Myoglobinemia	1	3	12-hourly	None stated	8% reduction in norepinephrine on day 1, 34% reduction on day 2
Akil et al. [75]	Septic shock, ARDS	13	2	24 h	30 day mortality	92.54% reduction in norepinephrine on day 1, 89% reduction on day 2, 100% reduction on day 3
Wallet et al. [76]	Cytokine release syndrome	1	2	24 h	None stated	100% reduction in norepinephrine on day 1
Mehta et al. [77]	Major aortic surgery	8	1	Intr-op use only	Changes in inflammatory markers	77% reduction in norepinephrine on day 1, 74% reduction on day 2
Alharthy et al. [78]	COVID-19, acute kidney injury	50	2	2 × 24 h	None stated	100% reduction in norepinephrine over 2 days in survivors
Rugg et al. [79]	Septic Shock	42	1	24 h	None Stated	52% reduction in norepinephrine on day 1, 54% reduction on day 2
Rieder et al. [80]	ARDS and ECMO	9	3	3 × 24 h	None Stated	37.5% reduction in norepinephrine on day 1, 50% day 2 and 100% by day 3.
Boss et al. [81]	Septic shock after cardiac surgery	98	1	24 h	None Stated	51.02% reduction in norepinephrine use, average use at least 15 h
